# Shortcut-to-Adiabaticity-Like Techniques for Parameter Estimation in Quantum Metrology

**DOI:** 10.3390/e22111251

**Published:** 2020-11-03

**Authors:** Marina Cabedo-Olaya, Juan Gonzalo Muga, Sofía Martínez-Garaot

**Affiliations:** Departamento de Química Física, UPV/EHU, Apdo 644, 48080 Bilbao, Spain; mcabedo001@ikasle.ehu.eus (M.C.-O.); sofia.martinez@ehu.es (S.M.-G.)

**Keywords:** shortcuts to adiabaticity, quantum metrology

## Abstract

Quantum metrology makes use of quantum mechanics to improve precision measurements and measurement sensitivities. It is usually formulated for time-independent Hamiltonians, but time-dependent Hamiltonians may offer advantages, such as a $T^{4}$ time dependence of the Fisher information which cannot be reached with a time-independent Hamiltonian. In *Optimal adaptive control for quantum metrology with time-dependent Hamiltonians* (Nature Communications 8, 2017), Shengshi Pang and Andrew N. Jordan put forward a Shortcut-to-adiabaticity (STA)-like method, specifically an approach formally similar to the “counterdiabatic approach”, adding a control term to the original Hamiltonian to reach the upper bound of the Fisher information. We revisit this work from the point of view of STA to set the relations and differences between STA-like methods in metrology and ordinary STA. This analysis paves the way for the application of other STA-like techniques in parameter estimation. In particular we explore the use of physical unitary transformations to propose alternative time-dependent Hamiltonians which may be easier to implement in the laboratory.

## 1. Introduction

Quantum metrology aims at high-resolution and highly sensitive measurements of parameters using advantages provided by quantum states and dynamics. Most of the research in this field has been focused on time-independent Hamiltonians, but time-dependent Hamiltonians may beat precision limits found for time-independent ones [1,2,3,4,5] to estimate some parameter *g* in the Hamiltonian.

The Cramèr–Rao bound states that the mean squared deviation $\langle \delta^{2}g\rangle$ for an unbiased estimation is bounded as

(1) $$\langle \delta^{2}g\rangle \geq \frac{1}{NI_{g}},$$

where *N* is a measure of the amount of data and $I_{g}$ is the Fisher Information.

In a quantum scenario the information about a parameter *g* in the Hamiltonian $H_{g}$ is “stored” in the quantum states of the system $|\psi_{g}\left(t\right)\rangle$, whose evolution depends on *g*, and the Fisher information for a final time *T* measures the distinguishability (distance) between $|\psi_{g}\left(T\right)\rangle$ and $|\psi_{g+\delta g}\left(T\right)\rangle$. The “maximum” Fisher information for *g* with respect to all possible quantum measurements is [6,7]

(2) $$I_{g}^{\left(Q\right)}=4\left(\langle \partial_{g}\psi_{g}\left(T\right)|\partial_{g}\psi_{g}\left(T\right)\rangle -{\left|\langle \psi_{g}\left(T\right)|\partial_{g}\psi_{g}\left(T\right)\rangle \right|}^{2}\right),$$

which is also named “quantum Fisher information”.

For a given Hamiltonian $H_{g}$, the quantum Fisher information has an upper bound,

(3) $$I_{g}^{\left(Q\right)}\leq {\left[\int_{0}^{T}\left(\mu_{max}\left(t\right)-\mu_{min}\left(t\right)\right)dt\right]}^{2},$$

where $\mu_{max}\left(t\right)$ and $\mu_{min}\left(t\right)$ are the (instantaneous) maximum and minimum eigenvalues of $\partial_{g}H_{g}\left(t\right)$. To actually implement this upper bound with particular states and measurements, the dynamics should follow some specific path, along an equal superposition of the corresponding eigenvectors. Reaching the upper bound of the Fisher information may require Hamiltonian control [1], i.e., adding an extra term $H_{c}\left(t\right)$ to the original Hamiltonian of the system $H_{g}\left(t\right)$ to implement the necessary dynamics. This methodology based on driving the system along preselected “rails” (states), is formally quite similar to the one proposed in Shortcut-to-adiabaticity (STA) methods [8], specifically in the “counterdiabatic” (CD) approach [9,10,11,12,13,14]. In the CD approach, an auxiliary Hamiltonian $H_{cd}\left(t\right)$ is also added to some reference Hamiltonian $H_{0}\left(t\right)$ to drive the system along eigenstates of $H_{0}\left(t\right)$. We shall revisit the main concepts and results in [1] in Section 2 and Section 3, and analyze in detail the relations and differences between “actual STA” and the STA-like method used in metrology, see Table 1 for an overview. A recurring topic within the counterdiabatic approach is that it is often difficult to implement in practice $H_{cd}$ [8,15,16]. This problem has lead to a number of approximations, variational approaches, or methods based on unitary transformations that, properly adjusted, could be as well applicable in metrology. In Section 4, we explore in particular the use of alternative Hamiltonians to $H_{g}+H_{c}$ via unitary transformations. In the final discussion, we shall comment on prospects to apply other STA-like approaches.

## 2. Optimal Adaptive Control for Quantum Metrology with Time-Dependent Hamiltonians

Our first objective is to summarize and comment on the work in [1] to set and understand the relations and differences between the STA-like approach applied there and ordinary STA. The analysis should be useful for a practitioner of STA methods less acquainted with quantum metrology, as well as for quantum metrologists not aware of the rich toolbox of STA techniques. In the following, ћ=1.

### Quantum Fisher Information

The quantum Fisher information in Equation (2) can be rewritten as (proportional to) a variance computed for the initial state $\psi_{0}$,

(4) $$I_{g}^{\left(Q\right)}=4\mathrm{Var}{\left[h_{g}\left(T\right)\right]}_{\psi_{0}},$$

where

(5) $$h_{g}\left(T\right)=i\;U_{g}^{†}\left(0\to T\right)\;\partial_{g}U_{g}\left(0\to T\right),$$

and $U_{g}\left(0\to T\right)$ is the unitary evolution operator from 0 to time *T* for the Hamiltonian $H_{g}\left(t\right)$. Being a variance, an “optimal” value of the quantum Fisher information, with respect to all possible initial states, is

(6) $$I_{g\;\;op}^{\left(Q\right)}\left(T\right)={\left[\tau_{max}\left(T\right)-\tau_{min}\left(T\right)\right]}^{2},$$

where $\tau_{max}\left(T\right)$ and $\tau_{min}\left(T\right)$ are maximal and minimal eigenvalues of $h_{g}\left(T\right)$, respectively. The optimal state $|\psi_{0}\rangle$ is an equal superposition of the maximal and minimal eigenvalues of $h_{g}\left(T\right)$, and the calculation of $I_{g\;\;op}^{\left(Q\right)}\left(T\right)$ requires diagonalizing $h_{g}\left(T\right)$. A mathematical upper bound for this optimal value may be found by rewritting $h_{g}\left(T\right)$ in integral form [1],

(7) $$h_{g}\left(T\right)=\int_{0}^{T}U_{g}^{†}\left(0\to t\right)\partial_{g}H_{g}\left(t\right)U_{g}\left(0\to t\right)dt.$$

The variance would be maximized by maximizing the contribution of each instant *t* by means of a hypothetical dynamical state that were at all times an equal superposition of the eigenvectors of $\partial_{g}H_{g}\left(t\right)$ with maximal and minimal instantaneous eigenvalues $\mu_{max}\left(t\right)$, $\mu_{min}\left(t\right)$,

(8) $$I_{g\;\;ub}^{\left(Q\right)}={\left[\int_{0}^{T}\left(\mu_{max}\left(t\right)-\mu_{min}\left(t\right)\right)dt\right]}^{2}.$$

We have termed this upper bound as “mathematical” because the eigenvectors $|\psi_{min}\left(0\right)\rangle$ or $|\psi_{max}\left(0\right)\rangle$ of $\partial_{g}H_{g}\left(0\right)$, with $\mu_{min}\left(0\right)$ and $\mu_{max}\left(0\right)$ eigenvalues, will not be driven in general along corresponding eigenvectors $|\psi_{min}\left(t\right)\rangle$ and $|\psi_{max}\left(t\right)\rangle$ with eigenvalues $\mu_{min}\left(t\right)$ and $\mu_{max}\left(t\right)$ by $H_{g}\left(t\right)$, i.e., in general the optimal value does not reach (saturate) the upper bound,

(9) $$\begin{array}{c} \tau_{max}\left(T\right)\leq \int_{0}^{T}\mu_{max}\left(t\right)dt,\;\;\;\tau_{min}\left(T\right)\geq \int_{0}^{T}\mu_{min}\left(t\right)dt. \end{array}$$

It is important to distinguish the quantum Fisher information in (4) (which is “maximal” with respect to measurements, for a given state), the “optimal” quantum Fisher information (6) (with respect to measurements and states), and the “upper bound” (8). The optimal value can be calculated in principle from the Hamiltonian $H_{g}\left(t\right)$ alone, but to implement it in an actual estimation protocol we would need specific states and measurements. Similarly, the upper bound depends formally only on $H_{g}\left(t\right)$ (more specifically on its derivative $\partial_{g}H_{g}\left(t\right)$), but its realization also needs careful state and measurement selection, as well as extra control terms, as we shall see. The terms “maximal”, “optimal”, and “upper bound” referred to the Fisher information represent here an ordered hierarchy but could be confusing and are subjected to specific defining conditions, they have to be put in context. It is not easy to keep an entirely consistent terminology, for example, “an optimal value” or an “upper bound” are of course also maximal values in some sense. Moreover Pang and Jordan refer to the process that achieves the upper bound (8) by adding Hamiltonian control as “optimal” [1]. Even the concept of an “upper bound” is a relative one as it depends on the chosen $H_{g}$. In summary, dealing with this somewhat entangled parlance needs careful reading.

Equation (7) gives the clue to physically realize the upper bound. Again, the initial state must be an equal combination of the maximal and minimal eigenvectors of $\partial_{g}H_{g}\left(0\right)$, and their time evolution should keep them as instantaneous eigenvectors of $\partial_{g}H_{g}\left(t\right)$ until t=T. The solution proposed in [1] to achieve this guided dynamics is to add a control term $H_{c}$ to the Hamiltonian $H_{g}$ so that the states are driven by the total Hamiltonian in the same way instantaneous eigenstates $|\psi_{k}\left(t\right)\rangle$ of $\partial_{g}H_{g}\left(t\right)$ change with time,

(10) $$H_{tot}\left(t\right)=H_{g}\left(t\right)+H_{c}\left(t\right).$$

Here is where the core similarity with STA (counterdiabatic) methods lays. Both in counterdiabatic methods and in the parameter estimation strategy set in [1], new terms are added to some reference Hamiltonian so that the system is guided along predetermined paths.

The proposed form of the control term is [1]

(11) $$H_{c}\left(t\right)=\sum_{k}f_{k}\left(t\right)|\psi_{k}\left(t\right)\rangle \langle \psi_{k}\left(t\right)|-H_{g}\left(t\right)+i\sum_{k}|\partial_{t}\psi_{k}\left(t\right)\rangle \langle \psi_{k}\left(t\right)|,$$

where the $f_{k}\left(t\right)$ are in principle arbitrary functions of time that could be chosen for convenience, and the $|\psi_{k}\left(t\right)\rangle$ are the instantaneous eigenvectors of $\partial_{g}H_{g}\left(t\right)$. Rewriting $H_{c}$ as

(12) $$H_{c}\left(t\right)=-H_{g}\left(t\right)+H_{cd}\left(t\right),$$

where

(13) $$H_{cd}\left(t\right)=\sum_{k}f_{k}\left(t\right)|\psi_{k}\left(t\right)\rangle \langle \psi_{k}\left(t\right)|+i\sum_{k}|\partial_{t}\psi_{k}\left(t\right)\rangle \langle \psi_{k}\left(t\right)|,$$

then

(14) $$H_{tot}\left(t\right)=H_{cd}\left(t\right).$$

Equation (13) may be found by *imposing* a unitary evolution operator of the form $U\left(0\to t\right)=\sum_{k}e^{-i\theta_{k}\left(t\right)}\left|\psi_{k}\left(t\right)\rangle \langle \psi_{k}\left(0\right)\right|$, which drives the dynamics along the eigenstates of $\partial_{g}H_{g}\left(t\right)$ up to phase factors $e^{-i\theta_{k}\left(t\right)}$. The corresponding Hamiltonian must be $i\dot{U}U^{†}$ which gives exactly the right hand side in Equation (13) with

(15) $$\theta_{k}\left(t\right)=\int_{0}^{t}f_{k}\left(t^{\prime }\right)dt^{\prime }.$$

In STA applications, $H_{cd}\left(t\right)$ is called counterdiabatic Hamiltonian [8] because, in that context, it avoids diabatic transitions among eigenstates of $H_{0}\left(t\right)$. $H_{cd}\left(t\right)$ drives the system along states $\left\{|\psi_{k}\left(t\right)\rangle \right\}$ both in STA applications and in metrology. There are, however, some important differences:

(i) In STA, the states $|\psi_{k}\left(t\right)\rangle$ are eigenstates of a reference Hamiltonian $H_{0}\left(t\right)$ (which plays a similar role than $H_{g}\left(t\right)$ as the Hamiltonian whose dynamics we want to transform by adding new terms) while in metrology they are eigenstates of $\partial_{g}H_{g}\left(t\right)$.

(ii) In STA, $H_{0}\left(t\right)$ is by construction diagonal in the basis $\left\{|\psi_{k}\left(t\right)\rangle \right\}$. In metrology $H_{g}\left(t\right)$ is in general not diagonal in this basis.

(iii) The functions $f_{k}\left(t\right)$ can be chosen to simplify the Hamiltonian. They do not produce transitions among the $\left\{|\psi_{k}\rangle \right\}$, they just accumulate a phase factor $e^{-i\theta_{k}\left(t\right)}$ for each $|\psi_{k}\left(t\right)\rangle$. In STA, we may apply this freedom to drive the system along the desired paths with $H_{tot}\left(t\right)=H_{0}\left(t\right)+H_{cd}\left(t\right)$, which is in fact the most common form, instead of $H_{tot}\left(t\right)=H_{cd}\left(t\right)$. By contrast, in metrology we could not in general use $H_{g}\left(t\right)+H_{cd}\left(t\right)$ because the addition of $H_{g}\left(t\right)$ does more than just changing phases, it produces transitions. That is why in metrology $H_{tot}\left(t\right)$ is just $H_{cd}\left(t\right)$, Equation (14), at least as a starting point, because a reformulation is in fact needed, see Equation (18) below and the related discussion.

(iv) In metrology the denomination “counterdiabatic” for $H_{cd}$ is, strictly speaking, an abuse of language as, in that context, $H_{cd}$ precludes transitions among eigenstates of $\partial_{g}H_{g}$, and not transitions among adiabatic states. Nevertheless, the formal expressions are identical so that we shall keep the same terminology and the same notation.

(v) The emphasis in STA is on fast processes, whereas in the STA-like approach used in metrology speed might be taken into account but it is not necessarily the main goal. Instead, the emphasis is on a precise parameter estimation.

Let us now come back to metrology. To recap, the addition of $H_{c}$ to $H_{g}$ would guarantee the state following needed in principle to reach the upper bound, but that is not enough, there are two very important points to take into account:

(a) Formally $H_{c}$ as written above, see Equation (11), depends on *g*, whose exact value is unknown. A way out is to set $H_{c}$ for an approximate value $g_{c}$.

(b) To get the upper bound of the Fisher information, in addition to following the *state* dynamics, the eigenvalues of $\partial_{g}H_{tot}$ should be the “right ones”, i.e., those of $\partial_{g}H_{g}$. This point is possibly not fully explicit in [1] but it is quite crucial, as the eigenvalues of $\partial_{g}H_{tot}$ for $H_{tot}=H_{cd}\left(g\right)$ are in general not the right ones.

The way out to these two points is to reformulate the control Hamiltonian in (11) as [1]

(16) $$H_{c}{\left(t\right)|}_{g\;=\;g_{c}}=\sum_{k}f_{k}\left(t\right)|{\tilde{\psi }}_{k}\left(t\right)\rangle \langle {\tilde{\psi }}_{k}\left(t)\right|-H_{g_{c}}\left(t\right)+i\sum_{k}|\partial_{t}{\tilde{\psi }}_{k}(t)\rangle \langle {\tilde{\psi }}_{k}\left(t)\right|,$$

where $|{\tilde{\psi }}_{k}(t)\rangle$ is the *k*th eigenstate of $\partial_{g}H_{g}\left(t\right)$ with $g=g_{c}$. In this context, the subscript $g=g_{c}$ does not mean that the value of $g_{c}$ is exactly equal to the unknown *g*. Rather, it means that $g_{c}$ should be written instead of the unknown *g*.

Instead of Equation (14), the total Hamiltonian is thus reformulated as

(17) $$H_{tot}\left(t\right)=H_{g}\left(t\right)+H_{c}{\left(t\right)|}_{g\;=\;g_{c}},$$

or, taking into account Equation (12),

(18) $$H_{tot}\left(t\right)=H_{g}\left(t\right)-H_{g}{\left(t)\right|}_{g\;=\;g_{c}}+H_{cd}{\left(t)\right|}_{g\;=\;g_{c}}=H_{g}\left(t\right)-H_{g_{c}}\left(t\right)+H_{cd}{\left(t)\right|}_{g\;=\;g_{c}}.$$

This is finally the structure used in Reference [1] to approach the upper bound of the Fisher information. The first term provides the right maximal and minimal eigenvalues of $\partial_{g}H_{g}\left(t\right)$, now $\partial_{g}H_{tot}\left(t\right)=\partial_{g}H_{g}\left(t\right)$, whereas the whole sum ($\approx H_{cd}\left(g\right)$ but not exactly) essentially drives the two corresponding eigenstates as dynamical solutions of the full Hamiltonian. This structure implies the need for an “adaptive scheme”, i.e., a guess value $g_{c}$ is taken as starting point to produce a better estimate $g_{c}^{\prime }$ and so on. Convergence towards *g* is not guaranteed for arbitrary circumstances, but in specific examples, the iterations do converge and convergence criteria may be found [1]. This motivates a further difference between ordinary STA and the STA-like approach:

(vi) The STA-like approach in metrology is adaptive, it proceeds by iteration to find via measurements, the value *g*. In ordinary STA there is no such a scheme, nothing plays the role of successive $g_{c},g_{c}^{\prime },g_{c}^{\prime \prime }...$ values. There are iterative approaches, such as the superadiabatic iterations [8], but their aim and formal structure do not match closely the described adaptive scheme. Nevertheless, superadiabatic iterations may be the basis for other parameter estimation schemes as sketched in the final discussion.

For the total Hamiltonian (18), Equation (7) is reformulated as

(19) $$h_{g}\left(T\right)=\int_{0}^{T}U^{†}\left(0\to t\right)\partial_{g}H_{tot}\left(t\right)U\left(0\to t\right)dt,$$

where $\partial_{g}H_{tot}=\partial_{g}H_{g}$ by construction; the unitary evolution operators U(0→t) and $U^{†}\left(0\to t\right)$ correspond now to the evolution driven by the total Hamiltonian $H_{tot}$. That is, the reformulated $h_{g}\left(T\right)$ depends both on *g* and $g_{c}$.

A further remark on notation: We stay essentially faithful to the compact notation in [1] to facilitate comparison, but compactness comes with a price as we use some symbols, for example $H_{tot}$ or $h_{g}$, for different things, contrast in particular (14) and the reformulation (18). A more precise but heavier notation would likely be cumbersome for the reader. We assume that the context should make clear the right interpretation. Note also that in the practical applications of the adaptive method in Reference [1] only the reformulated expressions for $H_{tot}$ and $h_{g}$ are used. In cases where doubts could arise, we will specify the equation number.

The eigenstates with the maximum and minimum eigenvalues of $\partial_{g}H_{g}{|}_{g\;=\;g_{c}}$ will be denoted as $|{\tilde{\psi }}_{max}(t)\rangle$ and $|{\tilde{\psi }}_{min}(t)\rangle$ respectively. With the initial state

(20) $$|\Psi (0)\rangle =\frac{1}{\sqrt{2}}\left(|{\tilde{\psi }}_{max}(0)\rangle +|{\tilde{\psi }}_{min}(0)\rangle \right),$$

the maximal Fisher information (2) reaches in zeroth order in the deviation the upper bound (8).

To attain in practice this upper bound of the quantum Fisher information, the following observable can be measured at time *T*,

(21) O=|+〉〈+|−|−〉〈−|,

where

(22) $$|\pm \rangle =\frac{1}{\sqrt{2}}\left(e^{-i\theta_{max}}|{\tilde{\psi }}_{max}(T)\rangle \pm e^{-i\theta_{min}}|{\tilde{\psi }}_{min}(T)\rangle \right).$$

Keeping dominant orders in $\delta g=g-g_{c}$,

(23) $$\begin{array}{ccc} \langle O\rangle & = & \cos(\delta g\int_{0}^{T}\left(\mu_{max}\left(t\right)-\mu_{min}\left(t\right)\right)dt), \end{array}$$

(24) $$\langle \Delta O^{2}\rangle =\sin^{2}\left(\delta g\int_{0}^{T}\left(\mu_{max}\left(t\right)-\mu_{min}\left(t\right)\right)dt\right),$$

so *g* can be found from the estimator 〈O〉. The variance of the estimate is the inverse of the upper bound of the Fisher information,

(25) $$\delta g^{2}=\frac{\langle \Delta O^{2}\rangle }{|\partial_{\delta g}{\langle O\rangle |}^{2}}=\frac{1}{{\left[\int_{0}^{T}\left(\mu_{max}\left(t\right)-\mu_{min}\left(t\right)\right)dt\right]}^{2}}.$$

## 3. Estimation of Field Amplitude and Rotation Frequency

Pang and Jordan [1] apply the above methodology to a qubit in a uniformly rotating magnetic field $B\left(t\right)=B[\cos\left(\omega t\right)e_{x}+\sin\left(\omega t\right)e_{z}]$, where $e_{x}$ and $e_{z}$ are unit vectors with directions $\hat{x}$ and $\hat{z}$, respectively, to estimate the amplitude *B* and the rotation frequency ω. Here, we shall focus on ω as it leads to the most interesting results.

The Hamiltonian that represents the interaction between the qubit and the field is

(26) $$H_{\omega }\left(t\right)=-B\left[\cos\left(\omega t\right)\;\sigma_{x}+\sin\left(\omega t\right)\;\sigma_{z}\right],$$

in terms of Pauli matrices. We may as well consider a reinterpretation of this Hamiltonian as the semiclassical interaction for a two level system in a properly set laser or microwave field, but let us keep formally the notation for a magnetic field.

An interesting exercise is to compute the Fisher information for ω (now the *g* parameter), with and without Hamiltonian control. The derivative of $H_{\omega }$ is

(27) $$\partial_{\omega }H_{\omega }\left(t\right)=tB\left[\sin\left(\omega t\right)\sigma_{x}-\cos\left(\omega t\right)\sigma_{z}\right],$$

with time-dependent eigenvalues $\mu_{max,min}=\pm tB$. Using this result in Equation (8), the upper bound of the Fisher information is

(28) $$I_{\omega \;ub}^{\left(Q\right)}=B^{2}T^{4}.$$

This result is nontrivial because the gap between Hamiltonian eigenvalues is not increased. Otherwise, if the Hamiltonian is set to increase rapidly with time, arbitrarily high powers of *T* or exponential growth may be found [1]. Note also that the maximum power of *T* that can be achieved with a time independent Hamiltonian is $T^{2}$.

Without Hamiltonian control the optimal quantum Fisher information (6) is instead

(29) $$I_{\omega ,0}^{\left(Q\right)}∼\frac{4B^{2}T^{2}}{4B^{2}+\omega^{2}}.$$

### Estimation of the Rotation Frequency with Hamiltonian Control

If we assume $f_{k}\left(t\right)=0$, (13) becomes

(30) $$H_{cd}=-\frac{\omega }{2}\sigma_{y}.$$

Note that $H_{cd}$ is here a time-independent Hamiltonian with an upper bound $∼T^{2}$ for the Fisher information. This illustrates the general statement made before that $H_{cd}$ drives along the “right” eigenvectors of $\partial_{g}H_{g}$ but does not necessarily provide the right eigenvalues as $\partial_{g}H_{cd}\neq \partial_{g}H_{g}$.

As explained in Section 2, the way out is to reformulate $H_{c}\left(t\right)$ at the estimated value $\omega_{c}$ and set

(31) $$\begin{array}{ccc} H_{tot} & = & H_{\omega }\left(t\right)+H_{c}{\left(t\right)|}_{\omega \;=\;\omega_{c}}\\ & = & -B\left[\cos\left(\omega t\right)\sigma_{x}+\sin\left(\omega t\right)\sigma_{z}\right]+B\left[\cos\left(\omega_{c}t\right)\sigma_{x}+\sin\left(\omega_{c}t\right)\sigma_{z}\right]-\frac{\omega_{c}}{2}\sigma_{y}. \end{array}$$

To compute the optimal Fisher information, the corresponding $h_{\omega }\left(T\right)$ in Equation (19) is diagonalized to find its eigenvalues. Since $\omega_{c}$ is assumed close to ω, $h_{\omega }\left(T\right)$ is expanded around $\omega_{c}=\omega$ as

(32) $$h_{\omega }\left(T\right)=h_{\omega }{\left(T\right)|}_{\omega_{c}\;=\;\omega }+\partial_{\omega_{c}}h_{\omega }\left(T\right){|}_{\omega_{c}\;=\;\omega }\delta \omega +\frac{1}{2!}\partial_{\omega_{c}}^{2}h_{\omega }\left(T\right)|\omega_{c}\;=\;\omega^{\delta \omega^{2}}+\ldots ,$$

where $\delta \omega =\omega_{c}-\omega$, (Notice that this notation in [1] is not consistent with $\delta g=g-g_{c}$.)

(33) $$h_{\omega }\left(T\right)=-\frac{BT^{2}}{2}\sigma_{z}-\frac{BT^{3}}{3}\sigma_{x}\delta \omega +\left(\frac{4B^{2}T^{5}}{15}+\frac{BT^{4}}{4}\sigma_{z}\right)\frac{\delta \omega^{2}}{2}+O\left(\delta \omega^{3}\right),$$

with eigenvalues

(34) $$\tau_{max,min}=\pm \frac{BT^{2}}{2}\mp \frac{BT^{4}}{72}\delta \omega^{2}+O\left(\delta \omega^{4}\right).$$

Therefore, substituting the results given by Equation (34) into Equation (6), the optimal Fisher information for (31) becomes

(35) $$\begin{array}{ccc} I_{\omega }^{\left(Q\right)} & = & B^{2}T^{4}\left(1-\frac{1}{18}T^{2}\delta \omega^{2}\right), \end{array}$$

where higher order terms of δω have been neglected. Conditions for convergence are analyzed in [1].

## 4. Alternative Driving via Physical Unitary Transformations

In ordinary applications of STA based on the counterdiabatic approach, $H_{cd}$ often implies different operators from those in the reference Hamiltonian $H_{0}$. These extra operators may be hard or even impossible to generate in the laboratory. In the applications to metrology of STA-like methods the same difficulties may arise with the control Hamiltonian $H_{c}$. Specifically, for the system and Hamiltonian studied in Section 3, the control Hamiltonian includes a $\sigma_{y}$ term whose implementation could be quite challenging in some systems [17], this really depends on the particular realization of the two-level system, but here we shall assume, as a basic exercise, that $\sigma_{y}$ is a term that we want to avoid. In STA applications, it is sometimes possible to change the structure of the total Hamiltonian avoiding undesired terms by means of “physical” unitary transformations [8,18,19]. We shall explore this approach in the context of parameter estimation. Specifically our generic goal is to modify the total Hamiltonian $H_{tot}$ (see Equation (18)) so that we get rid of the problematic terms. In the example of the previous section we will modify Equation (31), to get rid of the $\sigma_{y}$ term without losing the $T^{4}$ dependence in the Fisher information.

Given a Hamiltonian H(t) that drives the general state |ψ(t)〉, the unitarily transformed state $|\psi^{\prime }(t)\rangle =G^{†}\left(t\right)|\psi (t)\rangle$ obeys

(36) $$i\partial_{t}|\psi^{\prime }(t)\rangle =H^{\prime }\left(t)\right|\psi^{\prime }(t)\rangle ,$$

where

(37) $$\begin{array}{ccc} H^{\prime }\left(t\right) & = & G^{†}\left(t\right)\left[H\left(t\right)-K\left(t\right)\right]G\left(t\right), \end{array}$$

(38) $$\begin{array}{ccc} K(t) & = & i\dot{G}\left(t\right)G^{†}\left(t\right), \end{array}$$

and the dot stands for time derivative. $H^{\prime }\left(t\right)$ is in general not just the unitary transform of H(t) when G(t) depends on time. Notice also that although these expressions are formally the same as those that define an interaction picture, here the alternative Hamiltonian $H^{\prime }\left(t\right)$ and H(t) represent different physical drivings just like |ψ(t)〉 and $|\psi^{\prime }(t)\rangle$ represent different dynamic states. In the context of STA methods, the transformation provides indeed an alternative shortcut to the one represented by *H* if we set

(39) $$\begin{array}{ccc} G(0) & = & G(T)=1, \end{array}$$

(40) $$\begin{array}{ccc} \dot{G}\left(0\right) & = & \dot{G}\left(T\right)=0, \end{array}$$

in order to guarantee

(41) $$\begin{array}{c} |\psi^{\prime }(0)\rangle =|\psi (0)\rangle ,\;\;\;|\psi^{\prime }(T)\rangle =|\psi (T)\rangle , \end{array}$$

and

(42) $$\begin{array}{c} H^{\prime }\left(T\right)=H\left(T\right),\;\;\;H^{\prime }\left(0\right)=H\left(0\right). \end{array}$$

That is, with these boundary conditions the wavefunctions and the Hamiltonians coincide at the boundary times. In ordinary STA, these boundary conditions may be relaxed in some cases [19]. Moreover, in metrology as we shall see.

Let us now examine the operator $h_{g}^{\prime }=iU_{g}^{\prime †}\partial_{g}U_{g}^{\prime }$ corresponding to $H^{\prime }$, where

(43) $$\begin{array}{ccc} U_{g}^{\prime } & = & G^{†}U_{g}. \end{array}$$

The parameter *g* is unknown so we assume that the unitary transformation *G* does not depend on it. Then

(44) $$h_{g}^{\prime }\left(T\right)=iU_{g}^{\prime †}\partial_{g}U_{g}^{\prime }=iU_{g}^{†}\partial_{g}U_{g}=h_{g}\left(T\right).$$

Similarly $h_{g}^{\prime 2}\left(T\right)=h_{g}^{2}\left(T\right)$, so *H* and $H^{\prime }$ will have the same maximal Fisher information (four times the variance of $h_{g}$, see Equation (4)) for the same initial state. The optimal and upper bound Fisher information depend only on $h_{g}^{\prime }\left(T\right)=h_{g}\left(T\right)$ so they are also unaffected. In this context, there is no need in principle for the transformation operator *G* to satisfy the boundary conditions in Equations (39) and (40). However, it may be convenient to satisfy Equation (39) so that the wavefunctions |Ψ(t)〉 and $|\Psi^{\prime }\left(t\right)\rangle$ coincide at both initial and final times. In particular this would allow us to use the same observable *O* in Equation (21) as an estimator for *g*.

In the example of Section 3, we want to transform the Hamiltonian (31) to get rid of $\sigma_{y}$. We really need the full Hamiltonian (31) as starting point. If we instead used a pure $H_{cd}=-\frac{\omega }{2}\sigma_{y}$, as in Equation (14), only a $T^{2}$ dependence for the Fisher information could be reached since this Hamiltonian is time-independent.

When the Hamiltonian is a linear combination of generators of some Lie algebra, the unitary transformation *G* may be constructed by exponentiating elements of the algebra and imposing the vanishing of the unwanted terms [19]. In our example, the generators of the algebra are the Pauli matrices so, we will choose unitary transformations of the form

(45) $$G\left(t\right)=e^{-i\alpha \left(t\right)\sigma_{i}},$$

where α(t) is a given time-dependent real function and $\sigma_{i}$ can be any of the Pauli matrices $\{\sigma_{x}$, $\sigma_{y}$, $\sigma_{z}\}$. Taking into account that

(46) $$\begin{array}{ccc} e^{i\alpha \left(t\right)\sigma_{i}}\sigma_{i}e^{-i\alpha \left(t\right)\sigma_{i}} & = & \sigma_{i},\\ e^{i\alpha \left(t\right)\sigma_{i}}\sigma_{j}e^{-i\alpha \left(t\right)\sigma_{i}} & = & \cos\left[2\alpha \left(t\right)\right]\sigma_{j}+\frac{i}{2}\sin\left[2\alpha \left(t\right)\right]\left[\sigma_{i},\sigma_{j}\right],\;\;\;\;\;\;\;i\neq j, \end{array}$$

we choose $\sigma_{i}=\sigma_{y}$ in Equation (45). The alternative Hamiltonian becomes

(47) $$\begin{array}{ccc} H^{\prime }\left(t\right)= & - & B\left[\cos\left(\omega t+2\alpha \right)-\cos\left(\omega_{c}t+2\alpha \right)\right]\sigma_{x}\\ & - & \left[\frac{\omega_{c}}{2}+\dot{\alpha }\right]\sigma_{y}\\ & - & B\left[\sin\left(\omega t+2\alpha \right)-\sin\left(\omega_{c}t+2\alpha \right)\right]\sigma_{z}. \end{array}$$

To cancel the $\sigma_{y}$ term, we choose

(48) $$\alpha \left(t\right)=-\frac{\omega_{c}t}{2}.$$

(A similar unitary transformation is also used in [1] with different aim and results, see the Appendix A for further details.) Substituting Equation (48) into Equation (47) he have finally

(49) $$H^{\prime }\left(t\right)=B\left\{1-\cos\left[\left(\omega -\omega_{c}\right)t\right]\right\}\sigma_{x}-B\sin\left[\left(\omega -\omega_{c}\right)t\right]\sigma_{z},$$

which has the same structure (generators) as the reference Hamiltonian (26) but different time-dependent coefficients. Rewriting (49) as

(50) $$\begin{array}{ccc} H^{\prime }\left(t\right) & = & B\left[1-\cos\left(\omega t\right)\cos\left(\omega_{c}t\right)-\sin\left(\omega t\right)\sin\left(\omega_{c}t\right)\right]\sigma_{x}\\ & - & B\left[\cos\left(\omega_{c}t\right)\sin\left(\omega t\right)-\cos\left(\omega t\right)\sin\left(\omega_{c}t\right)\right]\sigma_{z}, \end{array}$$

it can be seen that the realization of $H^{\prime }\left(t\right)$ is possible assuming that fields oscillating with ω (a “carrier” signal with precise frequency to be determined) and $\omega_{c}$ (a test signal with accurately known frequency) can be implemented and combined. Alternatively, we may think of a setting where the difference between two frequencies $\omega -\omega_{c}$ can be controlled accurately even if the carrier frequency is unknown. Therefore, the alternative feasible Hamiltonian will keep the $T^{4}$ scaling of Fisher information for a given evolution time *T* and, consequently, the estimation of the ω will be the same than the one achieved using the untransformed Hamiltonian. Specifically an explicit perturbative calculation in orders of δω reproduces the result in Equation (35) in agreement with the general proof given above that the unitary transformation does not change the Fisher information.

As for the observable *O* in Equation (21), it may be used as an estimator provided G(0)=G(T)=1. Then the final states for drivings by *H* and $H^{\prime }$ are identical if the initial states are the same. Equation (48) implies that G(0)=1. Noting that, generically,

(51) $$\exp\left[\pm i\eta \left(t\right)\sigma_{i}\right]=\cos\left[\eta \left(t\right)\right]I\pm i\sin\left[\eta \left(t\right)\right]\sigma_{i},$$

at periodic times

(52) $$T=2n2\pi /\omega_{c},$$

G(T)=1 as desired.

## 5. Discussion

The seminal work of Pang and Jordan in [1] demonstrates that time-dependent Hamiltonians allow for better parameter estimations than time-independent ones. Specifically, the time dependence of the Fisher information can be given by higher powers of time without increasing Hamiltonian intensity. In the example of a qubit in a rotating magnetic field, the optimal Fisher information for the rotating frequency of the field can reach a $T^{4}$ dependence surpassing the limit $T^{2}$ of time-independent Hamiltonians. In practice, it is necessary in general to add a control Hamiltonian to reach the upper bound of the Fisher information. Pang and Jordan propose a control Hamiltonian to reach the upper bound using an STA-like adaptive approach.

We have discussed similarities and differences between actual STA methods and the STA-like method in Reference [1]. This analysis sets the ground to apply other STA techniques in metrology. We have explored here one of them: physical (rather than formal) unitary transformations. We have first proven that for these transformations the Fisher information does not change. Then, a “proof of principle” application is worked out for the frequency measurement in the single qubit model: assuming that a $\sigma_{y}$ type of term is not easy to implement, as it happens, e.g., in the experimental setting in [17], we find, by a unitary transformation, alternative Hamiltonians leading to the upper bound of the Fisher information without a $\sigma_{y}$ term.

As for further possibilities, we sketch here some ideas to be developed in more detail elsewhere: The counterdiabatic approach may be regarded as the zeroth iteration of an STA-generating scheme based on superadiabatic iterations [8,12,20,21]. In zeroth order a given (Schrödinger picture) Hamiltonian $H_{0}\left(t\right)$ is diagonilized with a basis $\left\{|n_{0}\left(t\right)\rangle \right\}$ to set an interaction picture (IP) based on the unitary transformation $A_{0}=\sum_{n}\left|n_{0}\left(t\right)\rangle \langle n_{0}\left(0\right)\right|$ with IP Hamiltonian $H_{1}=A_{0}^{†}\left(H_{0}-K_{0}\right)A_{0}$, where $K_{0}=i{\dot{A}}_{0}A_{0}^{†}$. If, in the IP, the coupling term is cancelled by adding its negative, $A_{0}^{†}K_{0}A_{0}$, the dynamics unfolds without transitions. Back in the Schrödinger picture (SP) this amounts to driving the system aided by a counterdiabatic term, with the modified Hamiltonian $H_{0}+K_{0}$, where $K_{0}=H_{cd}=H_{cd}^{\left(0\right)}$. The added superindex (0) denotes that higher order iterations may be worked out by repeating the same process, starting, in the first iteration, with $H_{1}$ instead of $H_{0}$. This first “superadiabatic” iteration generates a different coupling term that may be canceled with its negative as in the CD method. Of course further iterations could be implemented. The different iteration-dependent uncoupling terms $H_{cd}^{\left(j\right)}$ added to $H_{0}$ in the SP may or may not be useful depending on three main points [21]: (a) their “intensity” does not necessarily decrease with the iteration, typically it decreases first until it begins to grow [20]; (b) the operators involved in $H_{cd}^{\left(j\right)}$—in some operator basis—change with the iteration. Thus, some iterations may lead to undesired terms, difficult to implement, or, instead, to a convenient operator structure; (c) the higher order iterations, j≥1, beyond the CD method do not necessarily drive the system from $|n_{0}\left(0\right)\rangle$ to a final $|n_{0}\left(T\right)\rangle$, the eigenvectors of the original Hamiltonian, up to phase factors. For that end some boundary conditions should be satisfied, namely, that the successive $K_{j}$ vanish at the time boundaries [21]. In STA these conditions are satisfied by Hamiltonians $H_{0}$ with vanishing successive time derivatives [21,22].

To apply the above scheme to parameter estimation several changes are needed. First of all, the states $|n_{0}\left(t\right)\rangle$ should represent eigenstates of $\partial_{g}H_{g}\left(t\right)$ instead of eigenstates of the reference Hamiltonian $H_{g}$. Then, the SP for a given iteration cannot be $H_{g}+H_{cd}^{\left(j\right)}$ because $H_{g}$ is now not diagonal in $\left\{|n_{0}\left(t\right)\rangle \right\}$ in general. In addition, the fact that *g* is not accurately known should be taken into account. An adaptive reformulation is therefore needed similar to the one in [1]. Thus for an iteration *j*, instead of $H_{g}+H_{cd}^{\left(j\right)}$, the reformulated SP Hamiltonian must be of the form

(53) $$H_{g}-H{{|}_{g=g_{c}}+H_{cd}^{\left(j\right)}|}_{g=g_{c}},$$

and, moreover, the boundary conditions for the $K_{j}$ should be satisfied.

One more point to consider is that in STA, the spectral information of $H_{0}$ (eigenstates and eigenvectors) may not be easily available if at all. This is often the case in many-body systems. Therefore, building $H_{cd}$ with the usual recipe is not possible so several approximate techniques have been worked out that do not use spectral information [23,24,25,26]. These techniques might be applicable in parameter estimation protocols when the spectral information of $\partial_{g}H_{g}$ is not accessible. Other interesting open questions are the analysis of mixed, rather than pure, states [27], and looking for possible connections between the bound in Quantum Fisher information and quantum speed limits [28].

## Figures and Tables

**Table 1:** Main differences between the use of Shortcut-to-adiabaticity (STA)-like methodology in metrology, following the work in [1], and ordinary applications of STA (Counterdiabatic approach).

	STA	Metrology
$|\psi_{k}(t)\rangle$	Eigenstates of a reference Hamiltonian $H_{0}\left(t\right)$	Eigenstates of $\partial_{g}H_{g}\left(t\right)$
Reference Hamiltonian $H_{ref}=H_{0},H_{g}$	$H_{0}$ diagonal in the basis $\left\{|\psi_{k}\rangle \right\}$	$H_{g}$ not diagonal in the basis $\left\{|\psi_{k}|\right\}$
$H_{cd}$	Drives along adiabatic states of $H_{0}$	Drives along eigenstates of $\partial_{g}H_{g}\left(t\right)$ (so “CD” is here an abuse of language)
$H_{ref}+H_{cd}$	The addition of $H_{0}$ only changes phases $\theta_{k}\left(T\right)$	Adding $H_{g}$ would produce transitions so it is added AND subtracted, see (18)
Speed	Emphasis on Fast driving	Not necessarily fast
Iterations	“Superadiabatic”, structural changes	Adaptive, only the parameter changes

## Appendix A. Physical vs. Formal Transformations

In this Appendix, we compare the “interaction picture” transformation in the Supplementary Note 3 of [1] and the transformation in Section 4. While in both cases similar unitary operators, $\exp(-i\sigma_{y}\omega t/2)$ and $\exp(i\sigma_{y}\omega_{c}t/2)$, are applied, the aim, results, and physical content of the transformations are not the same. In [1], the two Hamiltonians involved are (26) and

(A1) $$-B\sigma_{x}+\omega \sigma_{y}/2.$$

Formally, (26) may be regarded as an interaction picture Hamiltonian of the “Schrödinger” Hamiltonian (A1) if the interaction picture wavefunction is defined in terms of the Schrödinger picture one as $\psi_{IP}=\exp\left(i\omega t\sigma_{y}/2\right)\psi_{S}$. As in ordinary applications of interaction pictures, the physics is the same in both pictures, they are just different representations of the same thing, and the aim of the transformation is to get a simple expression of the evolution operator driven by (26) making use of the time independent structure of (A1). In these equations only the exact value ω appears, and no control Hamiltonian has been added.

In Section 4, the starting Hamiltonian is instead (31), which is transformed via (37) using $G=\exp(i\omega_{c}t\sigma_{y}/2)$ into (49). The two Hamiltonians involved, (31) and (49), are different now from those in Reference [1], (26) and (A1). Moreover, the distinction between ω and $\omega_{c}$ plays a fundamental role, and the control Hamiltonian is added in (31). In Section 4, the two related Hamiltonians represent different physical settings and drive different dynamics. The transformation is now made to change the physics, it is not just a convenient, formal change of representation.
